# Supplementary material for: Bigmouth Buffalo Ictiobus cyprinellus sets freshwater teleost record as improved age analysis reveals centenarian longevity
Source: Commun Biol. 2019 May 23;2:197. doi: 10.1038/s42003-019-0452-0 (PMC6533251; doi:10.1038/s42003-019-0452-0)
Supplement: Supplementary file 2 — Reporting Summary [file 42003_2019_452_MOESM2_ESM.pdf]

nature research

Corresponding author(s): Alec Lackmann

Last updated by author(s): Apr 23, 2019

Reporting Summary

Nature Research wishes to improve the reproducibility of the work that we publish. This form provides structure for consistency and transparency in reporting. For further information on Nature Research policies, see Authors & Referees and the Editorial Policy Checklist.

Statistics

For all statistical analyses, confirm that the following items are present in the figure legend, table legend, main text, or Methods section.

n/a	Confirmed	
☒	☐	The exact sample size (*n*) for each experimental group/condition, given as a discrete number and unit of measurement
☒	☐	A statement on whether measurements were taken from distinct samples or whether the same sample was measured repeatedly
☐	☒	The statistical test(s) used AND whether they are one- or two-sided<br>*Only common tests should be described solely by name; describe more complex techniques in the Methods section.*
☒	☐	A description of all covariates tested
☒	☐	A description of any assumptions or corrections, such as tests of normality and adjustment for multiple comparisons
☐	☒	A full description of the statistical parameters including central tendency (e.g. means) or other basic estimates (e.g. regression coefficient) AND variation (e.g. standard deviation) or associated estimates of uncertainty (e.g. confidence intervals)
☐	☒	For null hypothesis testing, the test statistic (e.g. *F*, *t*, *r*) with confidence intervals, effect sizes, degrees of freedom and *P* value noted<br>*Give P values as exact values whenever suitable.*
☒	☐	For Bayesian analysis, information on the choice of priors and Markov chain Monte Carlo settings
☒	☐	For hierarchical and complex designs, identification of the appropriate level for tests and full reporting of outcomes
☐	☒	Estimates of effect sizes (e.g. Cohen's *d*, Pearson's *r*), indicating how they were calculated

Our web collection on statistics for biologists contains articles on many of the points above.

Software and code

Policy information about availability of computer code

Data collection	No software was used to collect data, but software was used to analyze data.
Data analysis	We used JMP Pro Statistical Discovery™ Software (Version 13.0, SAS Institute, Inc. 2014) and SigmaPlot (Version 11.2) for statistical analysis and graphical output.

For manuscripts utilizing custom algorithms or software that are central to the research but not yet described in published literature, software must be made available to editors/reviewers. We strongly encourage code deposition in a community repository (e.g. GitHub). See the Nature Research guidelines for submitting code & software for further information.

Data

Policy information about availability of data

All manuscripts must include a data availability statement. This statement should provide the following information, where applicable:

- Accession codes, unique identifiers, or web links for publicly available datasets
- A list of figures that have associated raw data
- A description of any restrictions on data availability

The data that support the findings of this study are available from the corresponding author upon reasonable request.

Field-specific reporting

Please select the one below that is the best fit for your research. If you are not sure, read the appropriate sections before making your selection.

☐ Life sciences ☐ Behavioural & social sciences ☒ Ecological, evolutionary & environmental sciences

Ecological, evolutionary & environmental sciences study design

All studies must disclose on these points even when the disclosure is negative.

Study description	Bigmouth Buffalo fish were collected from the field. We then quantified and validated their age and growth characteristics. The general design was to obtain as many fish (broadly distributed over their life history) as possible from as many locations as possible (see manuscript for more details).
Research sample	A sample of Bigmouth Buffalo were sought to reflect the natural population's demographic characteristics from each water body. The reference curves for bomb radiocarbon age validation were used from existing chronologies within North American freshwaters (from previous work done on other species; see manuscript for more details).
Sampling strategy	We tried to obtain 30 individuals (taken at random) from each population, but it was not always possible to obtain this many. Our sample sizes were sufficient in the sense that we obtained as many fish as we could.
Data collection	Fish were collected from the field, and ARL immediately began collecting relevant data in the field for all specimens obtained. In addition, EBL and MEC assisted data collection efforts on occasion. Additional data collection in the lab (i.e. otolith work) was conducted by ARL, and for the validation work AHA, ARL, and the National Ocean Sciences Accelerator Mass Spectrometry Facility at the Woods Hole Oceanographic Institution.
Timing and spatial scale	The catch-and-release study (photographing specimens' natural markings) began in 2011. Aging work did not begin until Fall of 2016. All data in all years were collected during the open water seasons (i.e. when the water bodies were not frozen over, ~April-October). Samples were taken from 12 populations across midwestern and southern Minnesota from four minor watersheds (two major drainages: Mississippi and Hudson Bay).
Data exclusions	The only data exclusions are for the growth analysis (Fig. 6) and for the bomb radiocarbon figure (Fig. 3), and both of these exclusions and the rationale are explicitly mentioned in the manuscript. For Fig. 6, the Minnetaga fish were excluded because these were young fish collected in the fall (explained more thoroughly in the text), and two other specimens that were unsexed. For Fig. 3, the Mississippi River drainage fish sample (n=1) was excluded (it is shown in Table 1), because it appears to exemplify a drainage difference compared to the Hudson Bay samples (all others (n=27)).
Reproducibility	Several populations were sampled for 2-3 field seasons (2016-2018), and for all of these, the same demographic signal was evident year after year.
Randomization	Bigmouth Buffalo were opportunistically sampled using a variety of sampling methods (see methods for details) to get as many fish as possible. The exception is the Artichoke Lake population in which a subsample of the commercial harvest was taken because logistically we could not take their entire harvest. For some populations (i.e. places where it became evident that 90% of individuals were over 80 years old), ARL conducted mark-recapture work in place of sacrificing more individuals for age determination.
Blinding	EBL and MEC were secondary and tertiary age reader's of thin-sectioned otoliths. These were scored independently without knowledge of the primary reader's (ARL) age score.

Did the study involve field work? ☒ Yes ☐ No

Field work, collection and transport

Field conditions	Field work was conducted during the open water season, outdoor conditions ranging from 1-30 Celsius from April to October.
Location	Samples were collected from 12 populations across midwestern and southern Minnesota from four minor watersheds (across two major basins: Mississippi and Hudson Bay drainages). These included 11 lakes and 1 site below a dam on a river. See methods for more details, and Fig. 8.
Access and import/export	An annual Minnesota State fishing license was used for fish collection via hook and line (and by bowfishers). Sampling permits for the use of nets were acquired via MEC at NDSU: Minnesota Department of Natural Resources Special Permit No. 21988 (Fisheries Research), Date 7 March 2017, and Special Permit No. 23775 (Fisheries Research), Date 2 May 2018 for:<br>Mark Clark, Ph.D. or designee<br>North Dakota State University (NDSU)<br>Dept. of Biological Sciences<br>NDSU Dept. 2715, P.O. Box 6050<br>Fargo, ND 58108-6050
Disturbance	Disturbances were minimized by scientifically utilizing every specimen that we could, including skeletons.

Reporting for specific materials, systems and methods

We require information from authors about some types of materials, experimental systems and methods used in many studies. Here, indicate whether each material, system or method listed is relevant to your study. If you are not sure if a list item applies to your research, read the appropriate section before selecting a response.

Materials & experimental systems

n/a	Involved in the study
☒	☐ Antibodies
☒	☐ Eukaryotic cell lines
☒	☐ Palaeontology
☐	☒ Animals and other organisms
☒	☐ Human research participants
☒	☐ Clinical data

Methods

n/a	Involved in the study
☒	☐ ChIP-seq
☒	☐ Flow cytometry
☒	☐ MRI-based neuroimaging

Animals and other organisms

Policy information about studies involving animals; ARRIVE guidelines recommended for reporting animal research

Laboratory animals

The study did not involve laboratory animals.

Wild animals

Bigmouth Buffalo females and males ranging from 2-112 years of age were captured in the field. Fish were caught by bowfishers, hook and line, gill net, fyke net, and seine. In cases where animals were not killed by the sampling method (e.g. by bowfishers, or by gill net), the fish were anesthetized and killed with MS-222. For fish that were not dissected in the field, fish were transported on ice and then dissected in the lab. For fish involved in mark-recapture, specimens were caught, tagged, photographed, and released in good condition in their natural habitat.

Field-collected samples

Otolith samples are stored dry at room temperature in lab drawers.

Ethics oversight

We have treated all animals in accordance with NDSU guidelines on animal care (IACUC protocol A17007).

Note that full information on the approval of the study protocol must also be provided in the manuscript.
